# Inflammatory and redox reprogramming of macrophages by HIV cell-to-cell transmission inhibits bone resorption capacity

**DOI:** 10.3389/fimmu.2025.1694065

**Published:** 2025-11-14

**Authors:** Franco A. Sviercz, Patricio Jarmoluk, Constanza Russo, Cynthia Alicia López, Nicole Freiberger, Cintia Cevallos, M. Victoria Delpino, Jorge Quarleri

**Affiliations:** Laboratorio de Inmunopatología Viral, Instituto de Investigaciones Biomédicas en Retrovirus y Sida (INBIRS), Universidad de Buenos Aires, Consejo Nacional de Investigaciones Científicas y Técnicas (CONICET), Buenos Aires, Argentina

**Keywords:** HIV, cell-to-cell transmission, osteoclast, bone resorption, redox imbalance, pyroptosis

## Abstract

**Introduction:**

People with HIV experience bone loss, but how viral spread perturbs osteoclastogenesis remains unclear. We asked whether cell-to-cell transmission of HIV from infected CD4^+^ T cells to macrophages reprograms precursors and impairs osteoclast differentiation.

**Methods:**

We co-cultured Jurkat cells infected with R5- or X4-tropic HIV with human monocyte-derived macrophages (M0/M1/M2) and quantified infection (p24/GFP), inflammasome activation and death (IL-1β, AnnexinV/7-AAD, z-YVAD), adhesion molecules/tetraspanins (ICAM-1, LFA-1, CD9/CD63/CD81), mROS (MitoSOX, NAC), polarization markers/cytokines, and osteoclastogenesis (TRAP, actin ring, CD51/61, adhesion, bone resorption).

**Results:**

R5 HIV infected M0>M2>M1 macrophages via contact, sustaining p24 release across differentiation and reducing TRAP^+^ osteoclasts and resorption. HIV-exposed macrophages showed inflammasome-linked death and IL-1β induction; contact enhanced Mf–T conjugates and upregulated ICAM-1/LFA-1 and tetraspanins. HIV-infected T cells displayed pro-inflammatory TNF-α/IFN-γ profiles, skewing macrophages toward M1-like states. Jurkat-derived ROS promoted conjugates and mROS accumulation in macrophages, while NAC reduced contact and oxidative imbalance. Nevirapine partially restored osteoclastogenesis and revealed contact-associated drug insensitivity.

**Discussion:**

The effects scaled with the proportion of infected T cells. HIV cell-to-cell spread induces inflammatory and redox reprogramming in macrophage precursors that blocks osteoclast differentiation and function, offering testable targets (inflammasome, adhesion, ROS) to protect bone in HIV.

## Graphical Abstract

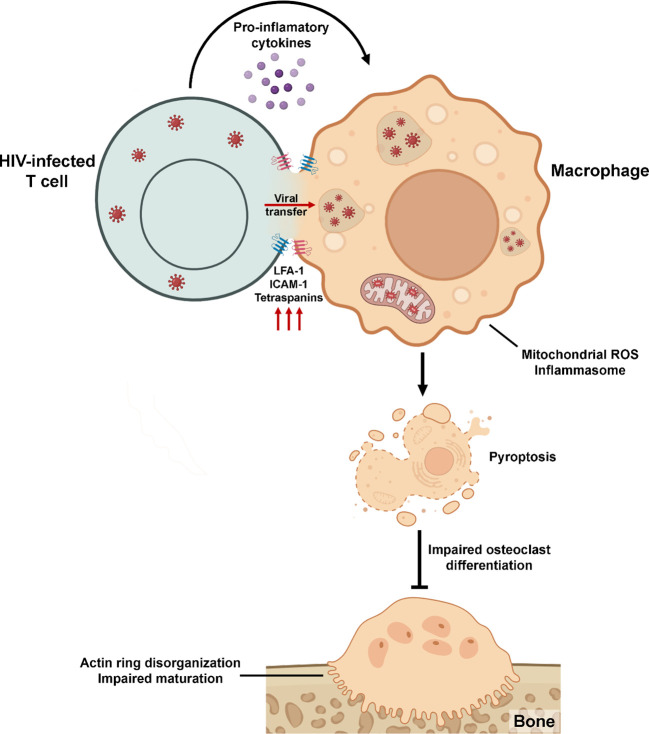

## 1 Introduction

Human Immunodeficiency Virus type-1 (HIV) infection remains a significant global health concern, not only because of its direct impact on the immune system but also due to its broader effects on non-lymphoid tissues, including the skeletal system. While CD4^+^ T lymphocytes have long been recognized as the primary targets of HIV (1), increasing evidence highlights the susceptibility of myeloid cells, particularly macrophages, to infection, especially through mechanisms involving direct cell-to-cell contact. This transmission mode enables the virus to evade immune surveillance and partially escape antiretroviral therapy (2).

Intercellular interactions between infected T lymphocytes and macrophages significantly enhance viral transfer efficiency. Several factors modulate this process, including viral tropism, the activation and differentiation status of the macrophages, and the relative abundance of infected donor cells (3–7). Despite growing recognition of the importance of these heterotypic contacts, the downstream consequences of macrophage HIV infection, particularly their differentiation into osteoclasts, remain insufficiently understood.

Osteoclastogenesis, the process by which macrophages differentiate into bone-resorbing osteoclasts, is critical for maintaining bone homeostasis. Disruption of this process has been implicated in the development of HIV-associated bone disorders, including osteopenia and osteoporosis (8–14). However, the specific mechanisms by which HIV infection alters osteoclast differentiation and function, particularly in the context of contact-mediated infection, are still poorly defined (8, 15).

However, some reviews suggest that chronic immune activation, changed cytokine levels, or imbalanced macrophage polarization in HIV could directly impair osteoclast differentiation. For instance, proinflammatory cytokines such as interferon-gamma (IFN-γ), which are frequently elevated in people living with HIV, can block RANKL signaling and osteoclast generation under certain conditions (16–22). These inhibitory effects are often context-dependent and may reflect systemic inflammatory states instead of direct suppression by HIV itself. Thus, the osteoclast compartment in HIV infection might face two influences: direct viral promotion of osteoclast activity and indirect inhibition through inflammatory pathways. Still, these observations mainly arise from broader immune and skeletal contexts, and there is limited evidence of a direct suppressive impact of HIV on osteoclast precursors.

Because macrophages are osteoclast precursors, their activation, viability, or differentiation alterations may contribute directly to HIV-associated bone disorders. Elucidating these mechanisms is essential for understanding the pathophysiology of HIV-induced bone loss and may reveal novel targets for therapeutic intervention.

## 2 Materials and methods

### 2.1 Monocyte isolation, differentiation, and Jurkat cells maintenance

Human monocytes were obtained from healthy donors and differentiated into monocyte-derived macrophages (Mf) following previously described methods (14). Each experiment included cells from a minimum of four biologically independent donors. Briefly, monocytes were purified (up to 80% purity) and seeded on slides in 24-well plates at 5 × 10^5^ cells/mL density in RPMI-1640 medium (Sigma-Aldrich, St. Louis, USA; cat. R8758) supplemented with 10% heat‐inactivated fetal bovine serum (FBS, Sigma‐Aldrich; cat. F7524), 2 mM of L‐glutamine (Gibco, Waltham, MA, USA; cat. 25030081), 1 mM of sodium pyruvate (Gibco; cat. 11360070), penicillin‐streptomycin (Gibco; cat. 15140122) (RPMI complete medium), and M‐CSF (10 ng/mL) (StemCell Technologies, Vancouver, BC, Canada; cat. 78150) for 6 days to generate Mf, serving as osteoclast precursors. To evaluate the effect of macrophage functional polarization on HIV infection efficiency and osteoclast differentiation, Mf at day 5 (a day before to coculture) were stimulated for 24 hours with lipopolysaccharide (LPS, 100 ng/mL; InvivoGen, San Diego, USA; cat. tlrl-eblps) to induce an M1 phenotype, with interleukin-4 (IL-4, 20 ng/mL; StemCell Technologies; cat. 78147) for an M2 phenotype, or left unstimulated as M0 controls.

Then, for osteoclast differentiation, Mf were further cultured in α-MEM medium (Gibco; cat. 11900073) supplemented with 10% FBS, 2 mM of L‐glutamine (Gibco; cat. 25030081), 1 mM of sodium pyruvate (Gibco; cat. 11360070), and penicillin‐streptomycin (Gibco; cat. 15140122) (α‐MEM complete medium), M-CSF (10 ng/mL; StemCell Technologies; cat. 78150), and RANKL (30 ng/mL; StemCell Technologies; cat. 78214) for 9 additional days (mature osteoclasts).

The human CD4^+^ T lymphocyte line Jurkat (Jk; clone E6.1), obtained from the American Type Culture Collection (ATCC, USA), was cultured in RPMI complete medium and maintained at 37°C in a humidified 5% CO_2_ incubator. The cells were routinely tested for mycoplasma contamination using the MycoAlert^®^ Mycoplasma Detection Kit (Lonza, Tampa, FL, USA; LT07-318).

### 2.2 HIV infection of macrophages via cell-to-cell transmission

Jurkat cells (Jk) were infected with HIV laboratory strains NLAD8-VSV (CCR5-tropic) or pNL43-GFP (CXCR4-tropic) using 0.5 pg of p24/cell via spinoculation as previously described (23) and cultured for 3 days. Infected or mock-infected Jk were washed, counted, and fluorescently labeled for cell tracking: VPD-450 (1 µM) (Violet Proliferation Dye 450; BD Biosciences, San Jose, CA, USA; cat. 562158) was used for all assays except adhesion/conjugate experiments, for which CFSE (0.5 µM) (5-(and-6)-carboxyfluorescein diacetate succinimidyl ester; TONBO Biosciences, San Diego, CA, USA; cat. 13-0850) was used. Labeled Jk cells were co-cultured with macrophages (Mf) at a 2:1 (Jk: Mf) ratio for 18 h to allow heterotypic cell-to-cell viral transmission. After coculture, non-adherent lymphocytes were removed by extensive PBS (Sigma-Aldrich; cat. D8537) washing, and macrophages alone (Mf-only) or exposed to Jk were cultured for 3 more days and then differentiated as described above. In selected experiments, viral replication was inhibited by adding Nevirapine (NVP, 1 µM; Sigma-Aldrich; cat. SML0097) before coculture.

To assess the effect of the number of infected Jk cells on both HIV infection efficiency and osteoclast differentiation, different ratios of NLAD8-VSV-infected Jk cells were mixed with non-infected Jk cells (10^-1^, 10^-2^, 10^-3^, and 10^-4^), and then co-cultured with Mf under the same conditions.

To assess soluble, contact-independent effects, we generated conditioned supernatants from HIV-R5–infected Jk cells (named as “St-R5”). Jk cells at 3 dpi were plated at the same cell density, in the same medium, and for the same 18-h incubation used for the co-culture condition but without macrophages. After 18 h, supernatants were collected, clarified, 0.45-µm-filtered, and added to macrophage cultures in place of Jk cells.

### 2.3 Measurement of HIV infection efficiency

HIV infection was assessed at 3 days-post-coculture (3 dpc) by intracellular p24 staining (KC57-PE; Beckman Coulter, Brea, CA, USA; cat. 6604667) for NLAD8 strain, after fixation and permeabilization with Cytofix/Cytoperm™ solution (BD Biosciences; cat. 554722), and by GFP detection for pNL43 strain using Full Spectrum Flow Cytometry Cytek^®^ Northern Lights 3000™ (Cytek Biosciences Inc.). Additionally, p24 levels in cell supernatants (NLAD8) were quantified by ELISA (Bio-Rad, Hercules, CA, USA; cat. 72386). For immunofluorescence, cells were seeded on glass coverslips (12 mm Ø; Paul Marienfeld GmbH & Co. KG, Lauda-Koenigshofen, Germany; cat. 0111520), fixed at 3 dpc in 4% paraformaldehyde (10 min, RT), permeabilized in Perm/Wash 1× and blocked with 1% BSA. Intracellular p24 (KC57-FITC; Beckman Coulter, cat. 6604665) and F-actin (Phalloidin-iFluor™ 532; Cayman Chemical, Ann Arbor, MI, USA; cat. 20551) were stained, nuclei were counterstained with DAPI, and coverslips were mounted on microscope slides (Paul Marienfeld GmbH & Co. KG; cat. 1000000) with Fluoromount-G™ (Thermo Fisher, cat. 00-4958-02). Confocal images were acquired on a Zeiss LSM 800 using an EC Plan-Neofluar 40×/0.75 air objective (pinhole 1 Airy unit; sequential channels); laser power and detector gain were kept constant within experiments. Images were analyzed in ImageJ/Fiji v2.16.0.

### 2.4 Assessment of macrophage cell death and inflammasome activation

Cell viability was assessed using the AnnexinV-APC/7-AAD apoptosis detection kit (BD Biosciences; cat. 559763) at 3 dpc through flow cytometry. Intracellular IL-1β production was detected by staining cells with an anti-IL-1β-PE antibody (BD Biosciences; cat. 340516) and anti-p24 (KC57-FITC, Beckman Coulter; cat. 6604665) after a 6-hour treatment with GolgiStop™ (BD Biosciences; cat. 554724) to inhibit cytokine secretion. Cells were then fixed, permeabilized, and analyzed by flow cytometry. Samples were acquired on a full-spectrum Cytek^®^ Northern Lights 3000™ spectral flow cytometer (Cytek Biosciences Inc.) using SpectroFlo. At least 30, 000 macrophage events after gating were recorded per sample, and event counts per condition were kept within ±10–15% to minimize sampling bias. Acquisition was performed at low–medium flow rate with an FSC threshold of 200, 000; detector gains were set by daily QC and kept constant within each experiment. Spectral unmixing was performed with single-stained reference controls and an unstained control; auto-fluorescence extraction was applied; FMO controls defined positivity. Replicates per condition are indicated in the figure legends. Detailed gating schemes are provided in the **Supplementary Data**.

To study the effect of proinflammatory cell death on cell viability, cocultures were pretreated with a pan-caspase-1 inhibitor, z-YVAD-fmk (50 μM; Sigma-Aldrich; cat. SML0429), for 1 hour before coculturing and were kept in that condition during the assay. The impact of Y-VAD treatment on cell viability was then assessed as described above.

### 2.5 Analysis of macrophage-T cell adhesion and surface receptors

For adhesion analysis, Jk cells were labeled with CFSE (0.5 μM, TONBO; cat. 13-0850), and Mf were labeled with VPD-450 (1 μM, BD Biosciences; cat. 562158) before coculture, and heterotypic conjugates were quantified by flow cytometry at 3 dpc. The impact of gp120-CCR5 interaction on cell-to-cell adhesion was evaluated by pre-treating cells with the CCR5 antagonist TAK-779 (3 µM; Sigma-Aldrich; cat. SML0911). Surface expression of adhesion- and fusion-related molecules was assessed by flow cytometry using the following fluorophore-conjugated antibodies: ICAM-1–FITC (BioLegend; cat. 353108), LFA-1–PE/Cy7 (BioLegend; cat. 363418), CD9–AF647 (BioLegend; cat. 124810), CD63–AF647 (BioLegend; cat. 353016) and CD81–APC (BioLegend; cat. 349510).

### 2.6 Analysis of cytokine gene expression in HIV-infected Jk cells

To analyze the effect of HIV infection (NLAD8 strain) on T cell gene expression, quantitative RT-qPCR was performed. Jk T cells were collected at 3 days post-infection and stimulated overnight with Phorbol 12-myristate 13-acetate (PMA, 20 ng/mL; Sigma-Aldrich; cat. P8139) and ionomycin (1 µM; Sigma-Aldrich; cat. I0634), as a non-specific activator.

Total RNA was extracted using the GenElute™ Total RNA Isolation Kit (Sigma-Aldrich; cat. AM1912), and RNA concentration was determined using a NanoDrop spectrophotometer (Thermo Fisher Scientific, Waltham, MA, USA). Complementary DNA (cDNA) was synthesized with the ImPromII Reverse Transcription System (Promega, Madison, WI, USA; cat. A3800), following the manufacturer’s protocol.

Quantitative real-time PCR was performed using SYBR-Select Master Mix (Applied Biosystems, Foster City, CA, USA; cat. 4472908) on a Bio-Rad qPCR system. The expression of IFN-γ, TNF-α, IL-13, and TGF-β was analyzed using the following thermal cycling conditions: 95°C for 5 minutes, followed by 42 cycles of 95°C for 5 seconds, 55°C for 15 seconds, and 72°C for 15 seconds.

Relative gene expression was calculated using the 2^–ΔΔCt method and normalized to GAPDH as a housekeeping gene. Intra-experimental Ct value variation between technical replicates was less than 0.5. Primer sequences used for amplification are listed in **Supplementary Table 1**.

### 2.7 Phenotypic analysis of macrophage polarization and activation

Macrophage activation state was determined by intracellular staining for TNF-α and IFN-γ at 0 dpc (immediately after the 18-h coculture), and by surface staining for CD80-PE (BioLegend; cat. 305208), CD86–PerCP/Cyanine5.5 (BioLegend; cat. 305420), CD206–APC (BioLegend; cat. 321110), and HLA-DR–PE-Cy5 (BD Biosciences; cat. 550475) at 3 days post-coculture (3 dpc), using Full Spectrum Flow Cytometry (Cytek^®^ Northern Lights 3000™, Cytek Biosciences Inc.). For intracellular cytokine detection, as previously mentioned, cells were treated with GolgiStop™ (BD Biosciences; cat. 554724) and fixed/permeabilized using Cytofix/Cytoperm™ BD Biosciences; cat. 554722) before staining. Cytokine concentrations (IL-4, IL-10, TNF-α, IL-6, and IL-1β) in cell-free supernatants collected at 3 dpc were quantified by ELISA using human BD OptEIA kits: IL-4 (BD Biosciences; cat. 555194), IL-10 (BD Biosciences; cat. 555157), TNF-α (BD Biosciences; cat. 555212), IL-6 (BD Biosciences; cat. 555220), and IL-1β (BD Biosciences; cat. 557953).

### 2.8 Redox balance assessment during infection and osteoclastogenesis

Mitochondrial reactive oxygen species (mROS) levels were assessed in the co-cultured cells by staining with MitoSOX™ Red (5 μM; Thermo Fisher Scientific; cat. M36008) for 30 minutes at 37°C, followed by flow cytometry analysis. mROS production was evaluated at multiple time points throughout the osteoclast differentiation process.

To evaluate the relative contribution of each cell type to the total mROS levels in heterotypic conjugates, a general ROS scavenger, N-acetyl-L-cysteine (NAC, 5 mM; Cayman Chemicals; cat. 20261), was selectively added either to Jk cells, to macrophages, or to both cell types. NAC treatment was applied for 5 hours before initiating the co-culture, followed by extensive washing to remove residual antioxidant.

### 2.9 Assessment of osteoclast differentiation, maturation, and bone resorption activity

Osteoclast formation was evaluated by Tartrate-Resistant Acid Phosphatase (TRAP) staining (Sigma-Aldrich; cat. 387A), following the manufacturer’s instructions. TRAP^+^ multinucleated cells (≥3 nuclei) were considered mature osteoclasts. TRAP-positive cells were imaged under an inverted microscope at 200× magnification (ECLIPSE TS100, Nikon) and counted using ImageJ software (version 2.16.0).

Cytoskeletal organization was assessed by F-actin staining using phalloidin-iFluor™ 532 (Cayman Chemical; cat. 20551), and visualized by confocal microscopy (Zeiss LSM 800, Zeiss, Germany).

Surface expression of the osteoclast integrin αVβ3 (CD51/CD61) was measured by flow cytometry using anti-CD51/61–FITC (BioLegend; cat. 304404). Osteoclast adhesion to plastic surfaces was evaluated as previously described (14). Briefly, cultures were washed with PBS 1× and incubated for 10 min at 37°C with Accutase^®^ (StemCell Technologies, cat. 07922) or, in parallel, with PBS 1× (control). After discarding the supernatant, remaining adherent cells were fixed in 4% paraformaldehyde (10 min, RT) and nuclei were stained with DAPI (Sigma-Aldrich, cat. D9542; 15 min). Five random fields per well were imaged at 200× on a Nikon ECLIPSE TS100 under identical illumination/exposure. Nuclei were counted in Fiji/ImageJ v2.16.0 using a fixed workflow. The adhesion index was calculated as the ratio of retained nuclei after Accutase to total nuclei in PBS-treated wells (normalized so that PBS=1).

Osteoclast function was assessed by resorption assays. Osteoclast precursors were seeded onto bovine cortical bone slices (Boneslices Inc., Jelling, Denmark; 6 mm Ø; 0.2/0.4 mm) and cultured with M-CSF (10 ng/mL; StemCell Technologies; cat. 78150) and RANKL (30 ng/mL; StemCell Technologies; cat. 78214) for 9 days. Bone resorption pits were stained with toluidine blue (Biopack, Buenos Aires, Argentina; cat. 9368.1), visualized under a light microscope (ECLIPSE TS100, Nikon). Bone resorption pits were stained with toluidine blue (Biopack, Buenos Aires, Argentina; cat. 9368.1) and imaged in bright-field under transmitted white light on an epifluorescence microscope (Nikon ECLIPSE TS100) using an S Plan Fluor ELWD 20×/0.45 DIC N1 objective, with identical illumination/exposure across conditions. Images were analyzed in ImageJ/Fiji v2.16.0; spatial calibration to µm/pixel was applied. Pit boundaries were segmented by operator-guided planimetry (ROIs stored in the ROI Manager) and areas measured. Resorbed area (%) per field was computed as Σ (pit ROI areas)/total field area.

### 2.10 Statistical analysis

Data were analyzed using GraphPad Prism 7 (Software Inc., San Diego, CA, USA). The exact values of n (donors) can be found in the figure legends. Normality was assessed with the Kolmogorov-Smirnov test. Comparisons were made using paired/unpaired t-test, or non-parametric alternatives (Mann-Whitney or Wilcoxon). p-values < 0.05 were considered statistically significant (*p ≤ 0.05; **p ≤ 0.01; ***p ≤ 0.001; ****p ≤ 0.0001, ns p >0.05).

All experiments adhered to BSL-3 laboratory standards at INBIRS, with biological materials autoclaved and incinerated following institutional rules.

### 2.11 Ethical approval

Ethical approval for this study was granted by the institutional review board and local ethical committee (Number: RESCD‐2023‐872). Buffy coats from healthy donors, aged 18–60 with a balanced gender ratio, were sourced from Hospital de Clínicas “José de San Martín, “ Facultad de Medicina, Universidad de Buenos Aires. All human samples, obtained regardless of this study, were provided without personally identifiable information.

### 2.12 Supplementary material

The supplemental material includes four figures that provide data supporting the main findings. **Supplementary Figure 1** outlines the experimental timeline and flow cytometry gating strategy for assessing HIV infection and osteoclast differentiation. **Supplementary Figure 2** presents controls and detailed analyses of macrophage viability, IL-1β production, and HIV p24 expression after co-culture with infected T cells. **Supplementary Figure 3** describes the macrophage-T cell conjugates gating strategy and quantification to show improved cell-to-cell contact following HIV exposure. **Supplementary Figure 4** features high-resolution confocal images of osteoclast cytoskeletal structures, emphasizing disrupted actin ring formation in cells exposed to HIV. Together, these figures validate and expand on the conclusions in the main text.

## 3 Results

### 3.1 Macrophage infection by HIV via cell-to-cell contact depends on viral tropism, macrophage polarization, and impairs osteoclast differentiation

**Figure 1:**
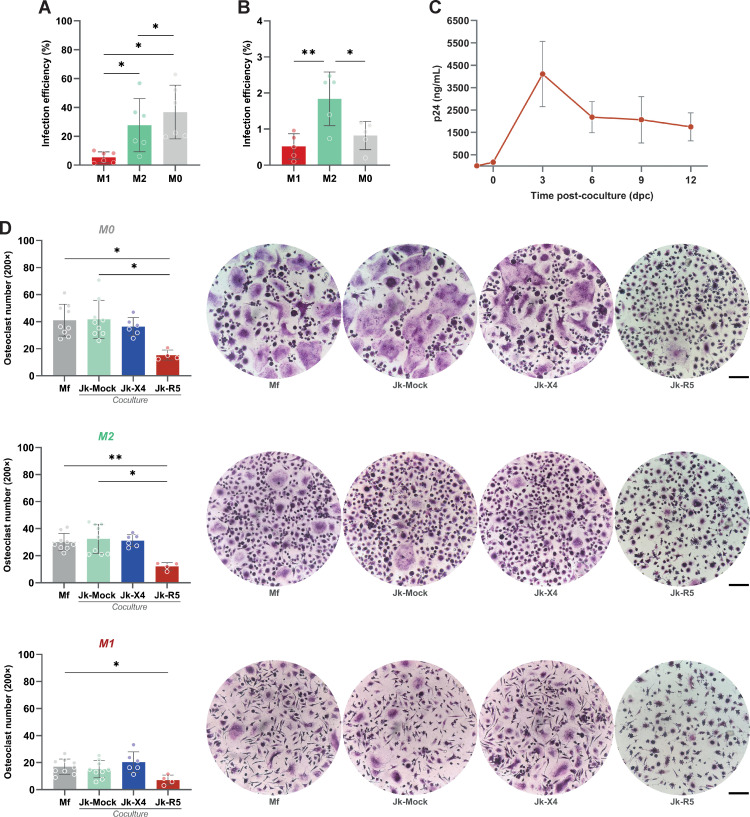
HIV infection via cell-to-cell contact is influenced by macrophage polarization, viral tropism, and impairs osteoclastogenesis. **(A)** HIV infection efficiency (% of p24^+^ macrophages) following Jk-R5 cells co-culture under different polarization states (M1, M2, M0). **(B)** HIV infection efficiency (% of GFP^+^ macrophages) following Jk-X4 cells co-culture under different polarization states (M1, M2, M0). **(C)** The kinetics of HIV replication were measured as p24 concentration (by ELISA) in a co-culture supernatant at indicated time points after contact with Jk-R5 cells (hpi = hours post infection). **(D)** Quantification and representative images (200×) of the number of TRAP-positive multinucleated osteoclasts (≥3 nuclei) at 12 dpc in each condition. Scale bar= 200 μm. Each dot represents a biological replicate. Data are presented as mean ± SEM. *p < 0.05, **p < 0.01.

To investigate the determinants of HIV transmission from infected CD4+ T lymphocytes to macrophages (Mf), PBMC-derived Mf were first polarized into M0, M1, or M2 phenotypes and then co-cultured with Jurkat (Jk) cells: mock-infected (Jk-Mock), infected with R5- (Jk-R5) or X4-tropic (Jk-X4) HIV strains (see experimental timeline in **Supplementary Figure S1A**), harboring comparable infection rate (25.5 ± 2.7% and 24.0 ± 6.4%, respectively; **Supplementary Figure S1B**). As shown in **Figures 1A, B**, flow cytometry analysis revealed that HIV infection efficiency varied depending on the viral tropism and macrophage activation state. M0 macrophages exhibited the highest permissiveness to cell-to-cell HIV-R5 infection (36.8 ± 18.6%), followed by M2 (27.7 ± 18.4%) and M1 (5.4 ± 3.8%). In contrast, X4-tropic HIV transmitted via infected Jurkat cells (Jk-X4) resulted in poor infection across all macrophage phenotypes, with infection levels remaining below 2%, even in M2 cells (1.8 ± 0.7%).

To determine whether HIV-infected macrophages support active viral replication over time, we quantified cell-free p24 in culture supernatants at serial time points from 0 dpc (immediately after the 18-h coculture) through 12 dpc, coinciding with the completion of osteoclast differentiation. The soluble p24 levels increased steadily, indicating that macrophages sustain productive HIV replication throughout the differentiation window (**Figure 1C**).

Functionally, heterotypic HIV infection mediated by cell-to-cell transfer impaired osteoclastogenesis. M-CSF/RANKL-induced differentiation of HIV-infected macrophages led to a marked reduction in TRAP^+^ multinucleated cells compared to uninfected controls (41.7 ± 14.1 cells/well). The extent of this impairment was shaped by both HIV tropism and the macrophage polarization state at the time of infection. M0 macrophages were particularly susceptible to R5-tropic HIV, exhibiting a pronounced decline in osteoclast formation (15.3 ± 3.8 cells/well), followed by M2-polarized cells (12.1 ± 2.7 cells/well). In contrast, M1-derived cultures formed few osteoclasts regardless of infection status (7.1 ± 3.6 cells/well), consistent with their intrinsically limited osteoclastogenic capacity (**Figure 1D**). X4-tropic HIV, delivered via infected Jurkat cells, did not significantly affect osteoclast formation in any polarization context.

These findings demonstrate that R5-tropic HIV, when transmitted via direct contact with infected lymphocytes, not only infects macrophages more efficiently but also disrupts their differentiation into osteoclasts in a polarization-dependent manner. This supports a model in which R5-mediated heterotypic cell-to-cell transmission compromises bone homeostasis through targeted impairment of osteoclastic differentiation, particularly affecting M0 and M2 macrophages.

### 3.2 Heterotypic HIV exposure compromises macrophage viability before osteoclast differentiation

**Figure 2:**
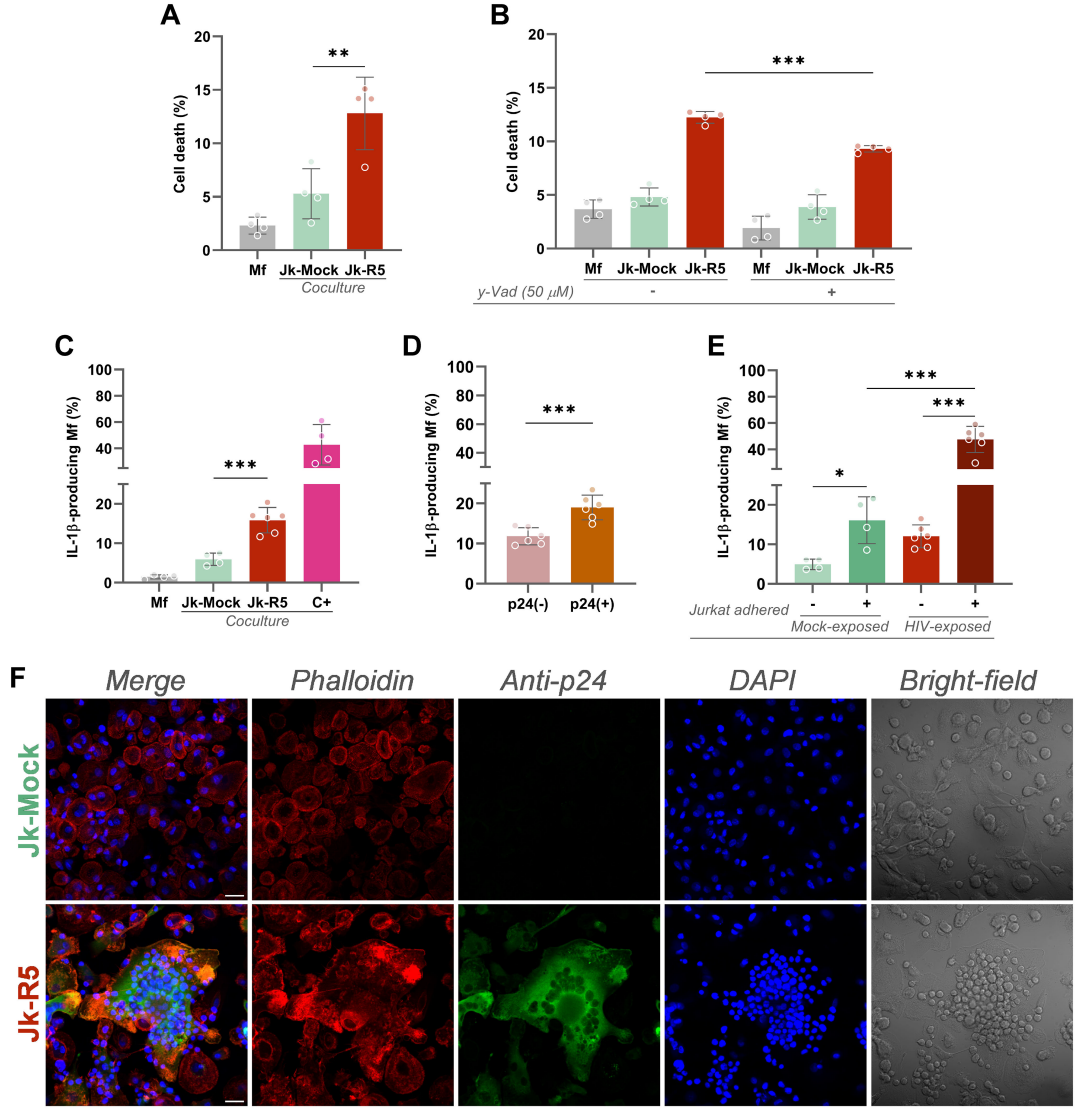
HIV-exposed macrophages undergo inflammasome-dependent cell death and IL-1β production. **(A)** Measurement of macrophage death (% AnnexinV^+^/7-AAD^+^) at 3 dpc following coculture with mock- or R5-infected Jurkat cells. **(B)** Effect of caspase-1 inhibition (z-YVAD-fmk, 50 μM) on cell death levels in each condition. **(C)** IL-1β-producing macrophages quantified by intracellular flow cytometry at 3 dpc. Positive control (C^+^): 18 h stimulation with LPS + ATP. **(D)** Frequency of IL-1β^+^ macrophages within p24^+^ and p24^-^ subsets in Jk-R5-exposed cultures. **(E)** Percentage of IL-1β^+^ macrophages exposed to mock- or R5-Jurkat cells, segregated by physical contact (contact vs. non-contact). **(F)** Representative confocal images showing phalloidin (red), p24 antigen (green), nuclei (DAPI, blue), and bright field. Scale bar = 25 μm. Each dot represents a biological replicate. Data represent mean ± SEM. *p < 0.05, **p < 0.01, ***p < 0.001.

To evaluate the effects of heterotypic co-culture on precursor cell viability, macrophages were exposed to Jk-R5 or Jk-mock cells for 72 hours before osteoclastogenic induction. As depicted in **Figure 2A**, flow cytometry analysis revealed a significant increase in regulated cell death in the Jk-R5-exposed group (12.8 ± 3.4%) compared to Jk-mock controls (5.3 ± 2.3%) and macrophage-only cultures (2.3 ± 0.8%). Jk viability remained stable throughout the experimental timeline, excluding confounding effects due to donor cell death (**Supplementary Figure S2A**).

Given the elevated levels of cell death, we investigated the involvement of pyroptosis by treating cocultures with the caspase-1 inhibitor z-YVAD-fmk (50 µM). Inhibition of caspase-1 activity led to a significant reduction in macrophage death, from 12.2 ± 0.6% to 9.3 ± 0.3% (**Figure 2B**), implicating inflammasome-dependent signaling in the observed cytotoxicity. These findings support a model in which direct contact with HIV-infected T cells triggers inflammasome activation and pyroptosis, contributing to macrophage depletion.

Consistent with inflammasome activation, the relative abundance (%) of IL-1β-producing macrophages was markedly increased in the Jk-R5 cocultures at 3 dpc (15.9 ± 2.6) compared to controls (5.9 ± 1.5) (**Figure 2C**). Among HIV-exposed macrophages, IL-1β production was significantly higher in p24^+^ (19.0 ± 3.1) than in p24^-^ cells (11.8 ± 2.1) (**Figure 2D**, **Supplementary Figure S2B**). As shown in **Figure 2E** and **Supplementary Figure S2B**, the highest levels were observed among infected macrophages physically interacting with Jurkat cells (47.6 ± 9.9), approaching values seen in the positive control (C^+^: 42.8 ± 15.4). These data underscore the contribution of productive infection and heterotypic cell contact to inflammasome activation.

Confocal microscopy further supported these findings, revealing that HIV-exposed macrophages displayed disrupted actin cytoskeleton integrity, as evidenced by altered phalloidin staining patterns (**Figure 2F**). Concurrent detection of intracellular HIV-p24 confirmed direct viral entry into macrophages. Moreover, HIV-exposed cells exhibited prominent and sustained adhesion to Jurkat cells, suggesting that cell-to-cell contact facilitates viral transmission and downstream activation.

Altogether, these results suggest that HIV cell-to-cell transfer via contact with infected lymphocytes allows viral entry and triggers early cellular stress and regulated cell death in macrophages. This compromised viability before RANKL stimulation may contribute to impaired osteoclast differentiation. These effects may be mediated by paracrine or contact-dependent signaling pathways initiated during heterotypic interaction.

### 3.3 HIV infection modulates adhesion molecule expression, influencing macrophage-lymphocyte contact

**Figure 3:**
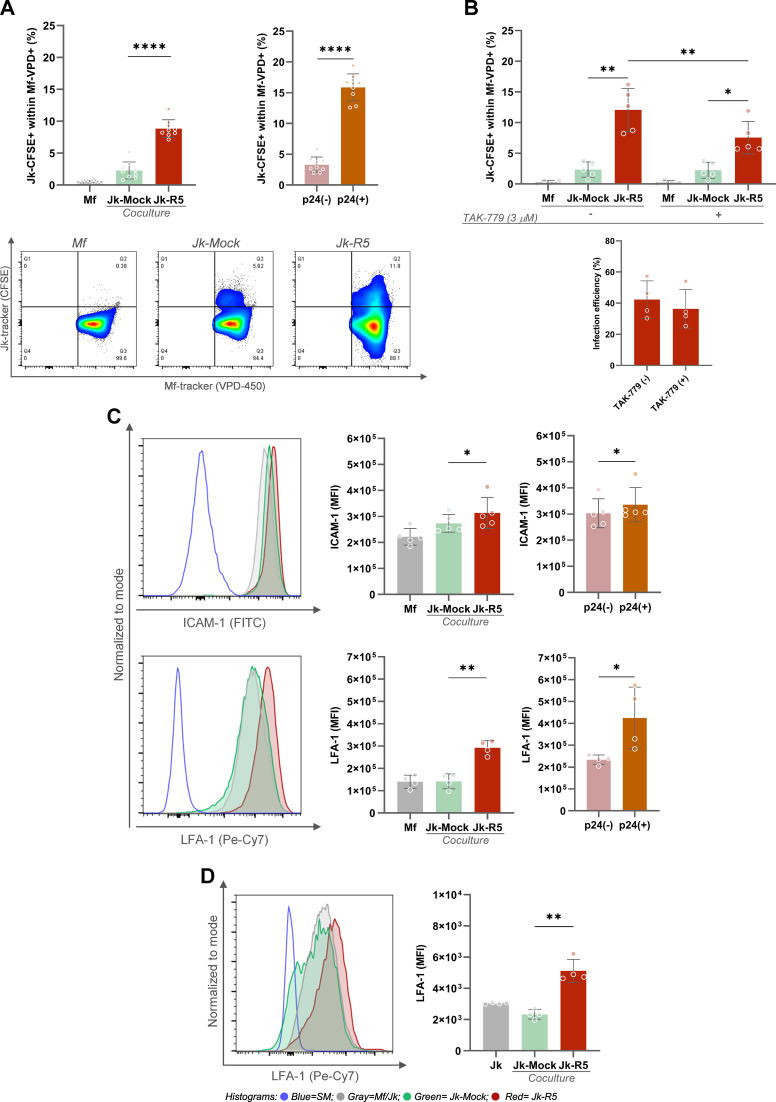
HIV exposure enhances macrophage-T cell conjugate formation and upregulates adhesion molecules. **(A)** Top left: Macrophages in physical contact with Jurkat cells frequency at 3 dpc following coculture, assessed by dual-tracker flow cytometry. Top right: Proportion of conjugates within p24^+^ and p24^-^ macrophage subsets in the Jk-R5 condition. Bottom: Representative dot plots showing macrophage–Jurkat conjugates in each condition. **(B)** Effect of CCR5 antagonism (TAK-779, 3 μM) on macrophage–Jurkat conjugate frequency (top) and HIV infection efficiency (bottom) at 3 dpc. **(C)** Top: Representative histograms and ICAM-1 expression (MFI) quantification in each condition and within p24^+^/p24^-^ macrophage subsets. Bottom: Histograms and quantification of LFA-1 expression (MFI) under the same conditions. **(D)** Representative histograms and LFA-1 expression (MFI) quantification in Jurkat cells after HIV infection. Each dot represents a biological replicate. Data are shown as mean ± SEM. *p < 0.05, **p < 0.01, ****p < 0.0001.

Efficient cell-to-cell transmission of HIV critically depends on stable intercellular contacts that facilitate viral synapse formation and direct viral transfer. A dual-label tracking approach was used to characterize the cellular interface underpinning viral transfer to evaluate the frequency of Mf–Jk conjugates (gating strategy **Supplementary Figure S3**). As shown in **Figure 3A**, the percentage of double-positive cell pairs (tracker overlap) increased from 2.3 ± 1.3 in non-infected cocultures to 8.9 ± 1.4 in the Jk-R5 condition at 3dpc. This indicates enhanced physical engagement, possibly through immunological synapse-like structures. Further analysis revealed that, among HIV-exposed macrophages, the highest levels of cell-cell adhesion occurred in those conjugates where the lymphocyte partner was effectively HIV-infected, as confirmed by intracellular p24 staining of the Jurkat cells (p24^neg^: 3.3 ± 1.2% vs. p24^pos^: 16.0 ± 2.2%; **Figure 3A**). This suggests that intercellular contact is favored or stabilized by a productive infection, thereby facilitating efficient viral transmission.

To further dissect the molecular mechanisms driving this enhanced cell-cell adhesion, we investigated the contribution of the viral gp120 envelope protein and its interaction with the cellular coreceptor CCR5, an interaction known to initiate virological synapse formation. To this end, we treated the co-cultures with the CCR5 antagonist TAK-779 before heterotypic contact. As shown in **Figure 3B**, the antagonism of CCR5 with TAK-779 significantly reduced the frequency of macrophage–lymphocyte conjugates, from 11.5 ± 3.7% in untreated HIV-exposed co-cultures to 7.4 ± 3.0% in the presence of the inhibitor. These findings indicate that the gp120–CCR5 interaction stabilizes intercellular adhesion and likely contributes to the efficiency of HIV cell-to-cell transmission. However, analysis of infection efficiency revealed that the percentage of HIV-p24^+^ macrophages remained comparable between TAK-779-untreated and treated groups (41.0 ± 14.4% vs. 37.9 ± 14.7% respectively; **Figure 3B**), suggesting that while CCR5 plays a role in enhancing adhesion, additional mechanisms beyond gp120–CCR5 interaction are involved in mediating viral entry and productive infection in macrophages.

Given the observed enhancement in cell-cell adhesion and the partial involvement of the gp120–CCR5 interaction, we examined the expression of key surface molecules known to mediate immune cell interactions and virological synapse formation. Adhesion molecules such as ICAM-1 and LFA-1 are critical for stabilizing cell-cell contacts. As shown in **Figure 3C**, surface profiling by flow cytometry revealed significant upregulation of ICAM-1 (x1.2-fold) and LFA-1 (x2.1) on macrophages exposed to Jk-R5. According to the infection status, both markers showed further upregulation in p24^+^ macrophages compared to their p24^-^ counterparts, with ICAM-1 increasing by 1.1-fold and LFA-1 by 1.8-fold. Jurkat cells also increased (x2.2-fold) the LFA-1 expression when HIV-infected (**Figure 3D**).

**Figure 4:**
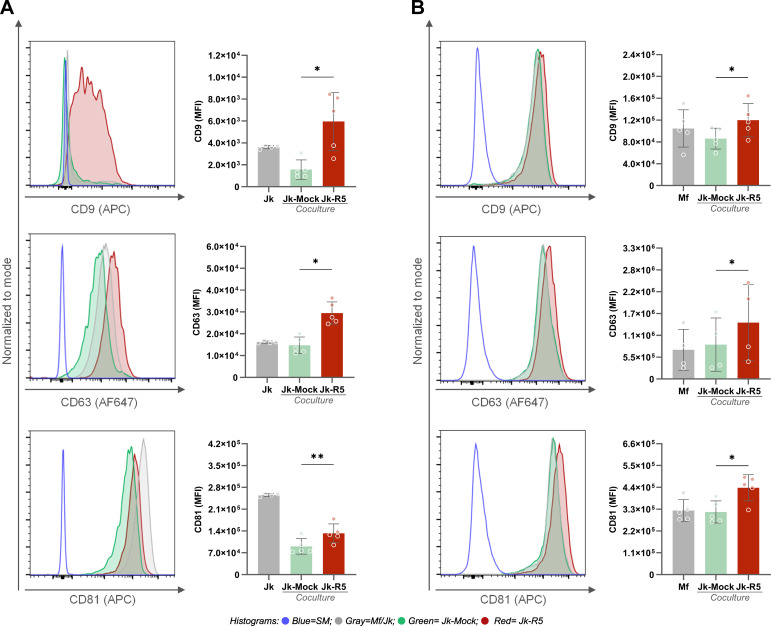
HIV exposure increases the expression of tetraspanins CD9, CD63, and CD81 in Jurkat cells and macrophages. **(A)** Representative histograms and quantification of CD9 (top), CD63 (middle), and CD81 (bottom) expression (MFI) on Jurkat cells after coculture with mock- or HIV-infected cells. **(B)** Representative histograms and quantification of CD9 (top), CD63 (middle), and CD81 (bottom) expression (MFI) on macrophages in the same conditions. Each dot represents a biological replicate. Data are presented as mean ± SEM. *p < 0.05, **p < 0.01.

The tetraspanin (CD9, CD63, and CD81) expression levels, measured by flow cytometry as median fluorescence intensity (MFI), were modulated by HIV infection and/or cell-to-cell contact. As shown in **Figure 4A**, among Jurkat cells, a significant upregulation of all three tetraspanins was observed in the Jk-R5 condition compared to mock-infected cells, suggesting that HIV infection and/or interaction with macrophages promotes tetraspanin expression in T cells. In macrophages (**Figure 4B**), a similar trend was observed: Mfs cocultured with HIV-infected Jurkat cells (Jk-R5) exhibited significantly higher expression of CD9, CD63, and CD81 compared to those cocultured with mock-infected Jurkat cells (Jk-mock). The p24^+^ macrophages depicted a higher CD81 expression than their p24^-^ counterparts, whereas no differences were observed for CD9 or CD63 (data not shown). It suggests that CD81 upregulation may be partially linked to productive infection, while the modulation of other tetraspanins could reflect broader effects of the coculture microenvironment. These results support a model where HIV appears to prime the intercellular junction, enhancing viral transfer and downstream signaling through sustained and stabilized cell-cell interactions.

### 3.4 HIV-infected T cells skew macrophage polarization toward a pro-inflammatory profile through cytokine modulation and direct contact

The interaction of T cells with macrophages can directly influence macrophage polarization and, osteoclast differentiation. Therefore, it is essential to characterize the cytokine expression profile of Jurkat cells at the time when intercellular contact is initiated.

**Figure 5:**
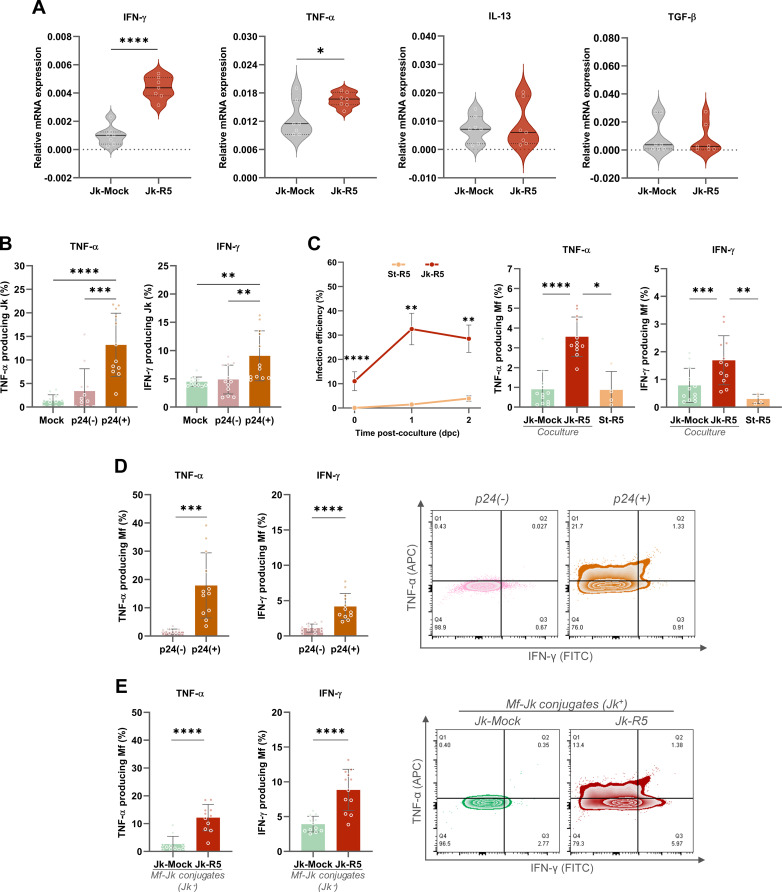
HIV-infected Jurkat cells promote a pro-inflammatory response in macrophages via cytokine modulation and cell-to-cell contact. **(A)** Relative mRNA expression of IFN-γ, TNF-α, IL-13, and TGF-β in Jurkat cells (Jk-Mock vs Jk-R5) at 3 dpi following PMA/ionomycin stimulation. The values of mRNA levels are relative to GAPDH (a housekeeping gene). **(B)** Frequency of TNF-α^+^ and IFN-γ^+^ Jurkat cells within p24^+^ and p24^-^ subsets after coculture (0 dpc). **(C)** Left: Kinetics of HIV-infection efficiency (measured by flow cytometry as cells expressing p24^+^) in macrophages after exposure to HIV-1 R5–infected Jurkat cells (red line: Jk-R5; contact condition) or to conditioned supernatants collected at 18 h from Jk-R5 cultures (orange line: St-R5; cell-free/soluble factors). Infection was assessed at 0, 1, and 2 days post-co-culture (dpc; 0 dpc ≡ 18 h from exposure) by intracellular p24 staining (clone KC57). and (center and right bars graphs) percentage of TNF-α^+^ and IFN-γ^+^ macrophages after coculture with mock- or HIV-infected Jurkat cells (Jk-uninfected vs Jk-R5) or Jk-R5-cell-free supernatants (St-R5). **(D)** TNF-α^+^ and IFN-γ^+^ macrophages relative frequency within p24^+^ and p24^-^ subsets (left), with representative contour plots (right). **(E)** Cytokine expression in macrophages in physical contact with Jurkat cells (Jk^+^ conjugates) in each coculture condition (left), with representative contour plots (right). Each dot represents a biological replicate. Data are presented as mean ± SEM. *p < 0.05, **p < 0.01, ***p < 0.001, ****p < 0.0001.

To assess this, we first evaluated the mRNA levels of pro- and anti-inflammatory cytokines (IFN-γ, TNF-α, IL-13, and TGF-β) in Jurkat cells at 3 days post-HIV infection (dpi), just before coculture. As shown in **Figure 5A**, infected Jurkat cells exhibited significantly increased transcription of IFN-γ (6.9-fold) and TNF-α (1.4-fold). In contrast, IL-13 (1.1-fold) and TGF-β (0.8-fold) expression levels remained unchanged, suggesting a shift toward a pro-inflammatory phenotype.

Given these observations, at 0 dpc (immediately after the 18-h coculture), the relative abundance (%) of TNF-α and IFN-γ producing Jurkat cells was measured by flow cytometry, including co-staining for intracellular p24. The relative abundance of HIV-infected (p24^+^) Jurkat cells reached 8.1 ± 3.5% (data not shown). The cytokine expression was predominantly enriched among the Jk-p24^+^ (TNF-α: 13.2 ± 6.7; IFN-γ: 9.1 ± 4.4), which exhibited significantly higher levels of TNF-α and IFN-γ than their p24^-^ counterparts (TNF-α: 3.4 ± 4.7; IFN-γ: 4.9 ± 2.6) and mock-infected controls (TNF-α: 1.4 ± 1.3; IFN-γ: 4.5 ± 0.8) (**Figure 5B**). These findings indicate that HIV-infected Jurkat cells display heterogeneous activation patterns, yet all share a pro-inflammatory phenotype that may contribute to the macrophage polarization observed in co-culture.

As shown in **Figure 5C**, HIV infection efficiency (% p24+) in macrophages exposed to St-R5 was markedly lower (p < 0.0001) than that observed in Jk-R5 co-cultures. At 0 dpc (immediately after co-culture), infection was undetectable in St-R5–exposed macrophages, whereas those in contact with Jk-R5 reached 11.1 ± 3.9% p24^+^. At 1 and 2 dpc, the infection efficiency among macrophages were: (i) exposed to Jk-R5 (1 dpc: 32.5 ± 6.5%; 2 dpc: 28.5 ± 5.6%) versus (ii) exposed to St-R5 (1 dpc: 1.5 ± 0.8%; 2 dpc: 3.9 ± 1.1%; p < 0.05). These macrophages in cell contact with HIV-Jurkat cells (Jk-R5) produced significantly more TNF-α (3.6 ± 1.0) and IFN-γ (1.7 ± 0.9) than those exposed to mock-infected Jurkat (TNF-α: 0.9 ± 1.0; IFN-γ: 0.8 ± 0.6) or even supernatant from HIV-infected Jurkat cells (TNF-α: 0.9 ± 0.9; IFN-γ: 0.3 ± 0.2), in which p24 levels remained undetectable. Furthermore, when discriminated within infected cocultures, the p24^+^ cell fraction produced even higher levels of TNF-α (17.9 ± 11.5) and IFN-γ (4.2 ± 1.9) than p24^-^ cells (TNF-α: 1.6 ± 0.9; IFN-γ: 1.1 ± 0.6) (**Figure 5D**).

Moreover, as shown in **Figure 5E**, when cytokine production was assessed discriminating between macrophages adhered to Jurkat cells and those that were not (Jk-R5: 14.1 ± 4.6 vs. Jk-Mock 10.1 ± 3.0 of adherent Jk to Mf; p ≤ 0.05), a marked increase in TNF-α (Jk-R5: 12.8 ± 5.0 vs Jk-Mock: 3.9 ± 5.2) and IFN-γ (Jk-R5: 8.8 ± 3.0 vs. Jk-Mock: 3.9 ± 1.1) levels were observed in infected cocultures compared with mock-exposed, suggesting that cell-to-cell contact with infected T cells is a key driver of inflammatory cytokine responses.

These findings highlight the importance of cell-to-cell contact in driving macrophage activation in the context of HIV exposure and underscore a crucial role for physical interaction between macrophages and infected T cells in triggering early immune activation.

**Figure 6:**
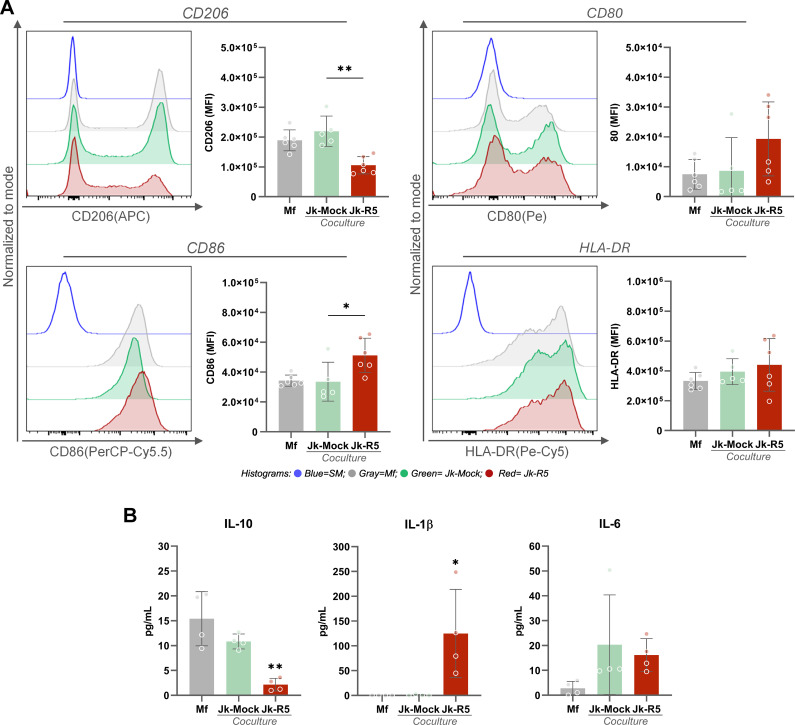
HIV-infected Jurkat cells skew macrophage polarization toward an M1-like profile. **(A)** Representative histograms and quantification of MFI by flow cytometry for CD206, CD80, CD86, and HLA-DR in macrophages at 3 dpc, following coculture with mock- or HIV-infected Jurkat cells. **(B)** IL-10, IL-1β, and IL-6 quantification levels in culture supernatants (pg/mL) at 3 dpc in the indicated conditions (measured by ELISA). Each dot represents a biological replicate. Data are presented as mean ± SEM. *p < 0.05, **p < 0.01.

Given the early immune activation, we further characterized macrophage polarization at 3dpc by profiling surface markers and cytokine production. As shown in **Figure 6A**, the expression level (MFI, ×10^5^) in the CD206 M2-associated marker among macrophages cultured alone (Mf), cocultured with mock-infected Jurkat cells (Jk-Mock), and those exposed to HIV-infected Jurkat (Jk-R5) cells exhibited a marked reduction (1.9 ± 0.3, 2.2 ± 0.5, and 1.1 ± 0.3, respectively). In contrast, the M1-associated marker CD86 was significantly increased (0.3 ± 0.04, 0.3 ± 0.1, and 0.5 ± 0.1). Both, CD80 (0.07 ± 0.05, 0.09 ± 0.1, and 0.2 ± 0.1) and HLA-DR (3.3 ± 0.6, 3.9 ± 0.9, and 4.3 ± 1.7) expression showed similar levels.

The level (pg/mL) of IL-10, IL-1β, and IL-6 released was measured in supernatants for these three conditions. As illustrated in **Figure 6B**, IL-10, a key anti-inflammatory cytokine associated with M2-like macrophage function, was significantly reduced in the HIV-Jk condition (Mf: 15.0 ± 5.4; Jk-Mock: 10.8 ± 1.5; Jk-R5: 2.2 ± 1.3). In contrast, the pro-inflammatory cytokine IL-1β was markedly elevated in Jk-R5 co-cultures (Mf: undetectable; Jk-Mock: 0.7 ± 1.4; Jk-R5: 124.9 ± 89.0), implying a strong inflammatory response. IL-6 levels were slightly lower in the HIV-Jk (Mf: 2.8 ± 2.8; Jk-Mock: 20.3 ± 20.1; Jk-R5: 16.2 ± 6.6), which may reflect a compensatory regulatory mechanism rather than a reduction in inflammation. At this time point, TNF-α, IFN-γ, and IL-4 were not detected in any tested conditions.

These findings collectively suggest that both direct contact and soluble mediators from HIV-infected T cells promote M1-skewed macrophage activation, contributing to the early pro-inflammatory environment characteristic of HIV infection. This immunological interplay may modulate the surrounding cytokine landscape to influence macrophage differentiation pathways, potentially leading to their transition into osteoclasts.

### 3.5 HIV impairs redox balance in macrophages during early and late osteoclastogenesis

The cellular redox status is a critical determinant of macrophage differentiation into osteoclasts and the dynamics of cell-cell interactions. Given the pro-inflammatory profile and pyroptotic signaling observed in macrophages upon contact with HIV-infected Jurkat cells, we investigated whether these interactions disrupt mitochondrial reactive oxygen species (mROS) levels in macrophages.

**Figure 7:**
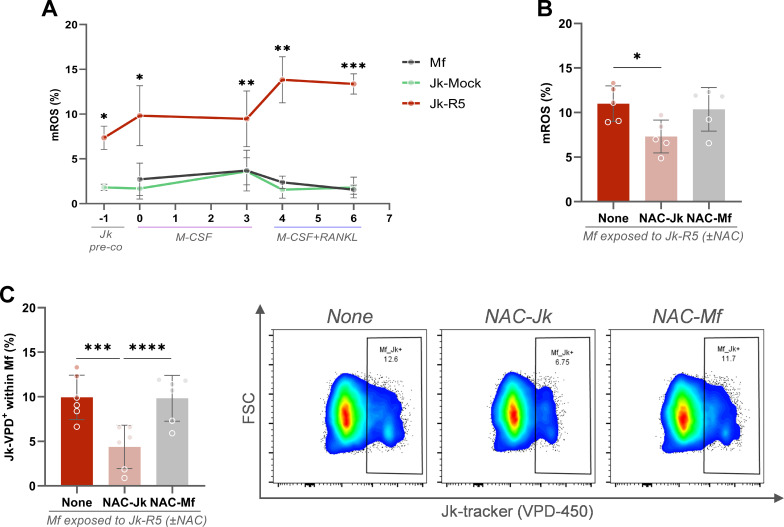
HIV-exposed macrophages exhibit sustained mitochondrial ROS production, and Jurkat-derived ROS promotes cell contact. **(A)** Kinetics of mitochondrial ROS (mROS) production (% MitoSOX^+^ cells) across different stages of osteoclast differentiation: Jurkat pre-coculture, pre-RANKL, and post-RANKL macrophages exposed to mock- or HIV-infected Jurkat cells. **(B)** mROS levels in macrophages at 0 dpc (immediately after the 18-h coculture) with HIV-infected Jurkat cells (Jk-R5), with or without N-acetylcysteine (NAC, 5 mM) treatment of Jurkat (NAC-Jk) or macrophages (NAC-Mf). **(C)** Frequency of macrophages in physical contact with Jurkat cells (Jk^+^ conjugates) at 0 dpc (immediately after the 18-h coculture) under the same NAC conditions. Representative dot plots (right) show gated events. Each dot represents a biological replicate. Data are shown as mean ± SEM. *p < 0.05, **p < 0.01, ***p < 0.001, ****p < 0.0001.

As shown in **Figure 7A**, the relative abundance (%) of mROS-producing cells among HIV-infected Jurkat cells (at 3 dpi, just before coculture) was significantly higher than in their mock-infected counterparts (7.4 ± 1.3 and 1.8 ± 0.4). This mROS imbalance persisted even during early osteoclast differentiation stages (days 4 and 6), after RANKL addition.

Before coculture, we used N-acetylcysteine (NAC, 5 mM), a potent antioxidant, to reduce intracellular mROS in Jurkat-R5 cells or macrophages, define its cellular source, and contribution to cell contact. As shown in **Figure 7B**, the abundance (%) of macrophages producing mROS was significantly reduced when cocultured with NAC-treated, HIV-infected Jurkat cells (Untreated: 11.0 ± 2.0; NAC-Jk: 7.3 ± 1.8). Thus, HIV-infected T cells appear to be a source of ROS, being rapidly transferred to macrophages during early interaction and, when neutralized, cell-to-cell contacts are markedly impaired (NAC-Jk: 4.4 ± 2.4). Nonetheless, neither their abundance (10.4 ± 2.5) nor their adhesion to Jurkat was altered when macrophages were pretreated with NAC (**Figure 7C**). These findings support a model in which ROS derived from HIV-infected T cells drive oxidative stress in macrophages and promote the formation of stable heterotypic conjugates, likely through the modulation of adhesion molecule activity.

### 3.6 HIV-driven impairment of osteoclast differentiation depends on viral replication and the degree of contact with infected T lymphocytes

**Figure 8:**
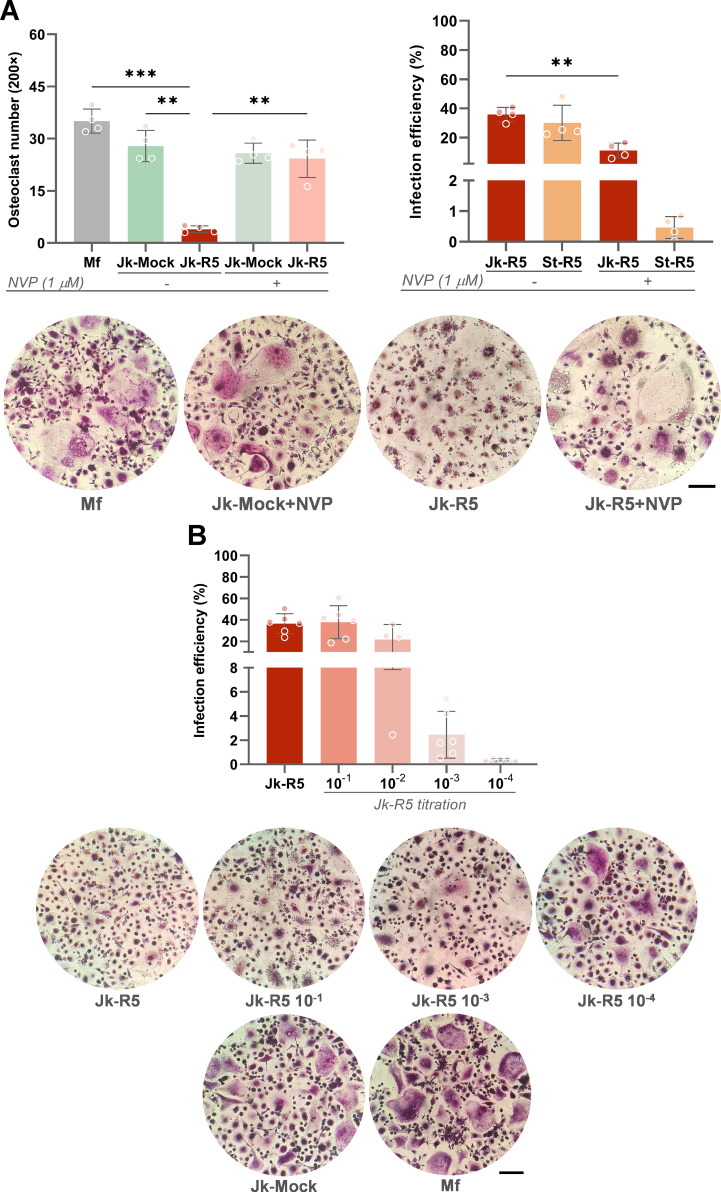
HIV impairs osteoclastogenesis in a replication- and dose-dependent manner. **(A)** Top-left: Osteoclast number quantification (TRAP^+^ multinucleated cells/well) at 12 dpc in the indicated cocultures, with or without nevirapine (NVP, 1 μM) treatment. Top-right: HIV infection efficiency (% p24^+^ macrophages) at 3 dpc measured by flow cytometry in Jk-R5 cocultures or cell-free supernatants, in the presence or absence of NVP. Representative TRAP staining images (200×) are shown below. **(B)** Top: Infection efficiency of macrophages after exposure to serial dilutions of HIV-infected Jurkat cells (Jk-R5). Bottom: Representative images of TRAP-stained osteoclasts for each condition (200×). Scale bar = 200 μm. Each dot represents a biological replicate. Data are shown as mean ± SEM. **p < 0.01, ***p < 0.001.

Building on the quantitative impairment established in Section 1, we next investigated the role of viral replication in macrophages in modulating osteoclast formation and function. As shown in **Figure 8A**, the HIV-induced impairment on osteoclastogenesis was reversed when the viral replication was inhibited using nevirapine (NVP, 1 µM) during the entire experimental schedule (Jk-R5: 3.9 ± 1.0 vs. Jk-R5+NVP: 24.3 ± 5.4 cells/well).

Notably, at 3 dpc, before RANKL addition, the NVP-efficiency to inhibit HIV-replication was dissimilar according to the route of macrophages’ HIV-infection. Hence, using cell-free virus, HIV-infection efficiency (%) in macrophages decreased drastically from 30.0 ± 12.1 to 0.5 ± 0.4. In contrast, the NVP-induced reduction was significantly lower, decreasing from 35.9 ± 4.8 to 11.1 ± 5.6 during cell-to-cell transmission (**Figure 8A**).

The perturbation of osteoclastogenesis resulting from cell-to-cell HIV infection of precursors exhibits a dose-dependent pattern. Titration assays showed that infection rates increased progressively with higher Jurkat-R5 to macrophage (Jk-R5:Mf) ratios in cocultures, reaching approximately 36.5 ± 9.2% p24^+^ macrophages at a 2:1 ratio and remaining detectable (~2.5 ± 1.9%) even at a 1:1, 000 ratio. These findings indicate that the degree of osteoclastogenesis inhibition correlates with the proportion of HIV-infected T cells in contact with macrophages, underscoring the critical role of both viral load and exposure level in determining the magnitude of the observed effect (**Figure 8B**).

**Figure 9:**
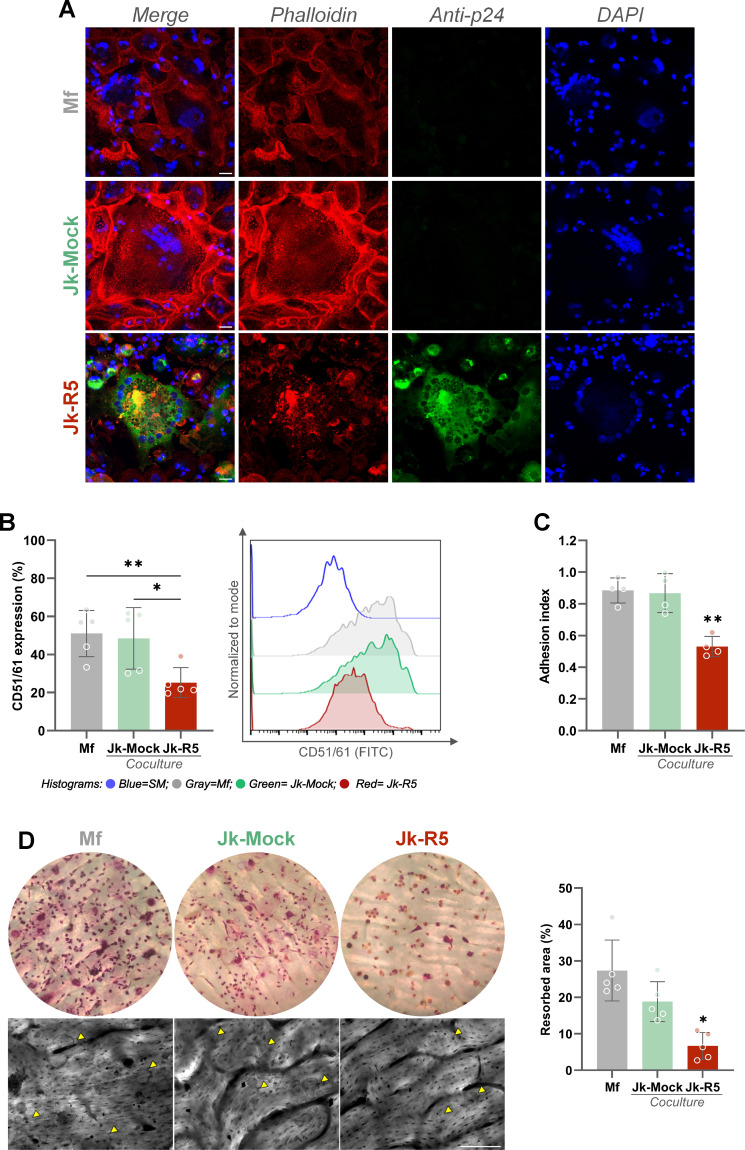
HIV impairs osteoclast function by disrupting cytoskeletal organization, integrin expression, adhesion, and resorption capacity. **(A)** Representative confocal images of osteoclast precursors exposed to mock- or HIV-infected Jurkat cells (Jk-R5) at 12 dpc. Cells were stained for F-actin (phalloidin, red), HIV p24 antigen (green), and nuclei (DAPI, blue). **(B)** Surface expression of the osteoclast-specific integrin CD51/61 measured by flow cytometry. Left: percentage of CD51/61^+^ cells; Right: representative histogram overlay. **(C)** Adhesion index of osteoclasts to plastic surface across coculture conditions. **(D)** Bone resorption capacity evaluated on dentine slices. Left: representative images of osteoclasts stained for TRAP on bone slices (top) and corresponding resorption pits visualized by toluidine blue (bottom, yellow arrowheads). Right: quantification of resorbed area (%). Scale bars = 25 μm **(A)**, 50 μm **(D)**. Each dot represents a biological replicate. Data are shown as mean ± SEM. *p < 0.05, **p < 0.01.

Beyond the quantitative reduction in osteoclast numbers, our results also reveal a qualitative disruption of osteoclast function. As shown in **Figure 9A**, confocal microscopy with phalloidin staining revealed disrupted cytoskeletal architecture, with actin rings—structures essential for bone adhesion and resorption—being absent or severely disorganized in HIV-exposed cells (see also **Supplementary Figures S4A, B**).

Consistently, when comparing the osteoclast maturation (measured by the integrin CD51/61 relative expression) between those obtained from macrophages cocultured with Jk-Mock and those with Jk-R5, a significant reduction was measured (48.4 ± 16.2 and 25.2 ± 7.9; **Figure 9B**). Besides, the cellular adherence (as an index) to inorganic matrix also showed a decrease under HIV infection: 0.9 ± 0.1 vs. 0.5 ± 0.1 (**Figure 9C**). Lastly, a markedly reduced bone resorption capacity was also measured, reflected in fewer and smaller resorption pits (Mf: 27.4 ± 8.4; Jk-Mock: 18.8 ± 5.5; Jk-R5: 6.7 ± 3.7%) (**Figure 9D**).

Together, these findings indicate that HIV not only limits the number of osteoclasts in a replication- and dose-dependent manner but also compromises the functional capacity of the osteoclasts that do form, pointing to a multifaceted mechanism by which HIV disrupts bone homeostasis.

## 4 Discussion

This study demonstrates that the HIV transmission from infected T lymphocytes to macrophages significantly disrupts the formation of osteoclasts. These interactions, which occur through inflammatory and redox pathways, interfere with the progression of macrophages into functional osteoclasts.

Cell-to-cell transmission spreads the virus more effectively than cell-free infection. It allows HIV to avoid neutralizing antibodies and some antiretroviral drugs (3, 4, 7, 24–26). In our study, this mode of transmission was also more efficient at infecting unpolarized (M0) and alternatively activated (M2) macrophages, subsets known to be more permissive to HIV replication because of their lower restriction capacity and altered expression of viral entry receptors (1, 5). In accordance, in our model, although nevirapine substantially reduced HIV infection in macrophages exposed to cell-free virus, its inhibitory effect was markedly attenuated during cell-to-cell transmission, where the infection may persist. This observation supports the notion that the confined microenvironment of the virological synapse may shield the virus from drug access, contributing to residual infection despite antiretroviral therapy. Such findings have critical implications, as they suggest that direct contact between HIV-infected T cells and macrophages in tissue niches could sustain local viral reservoirs and mediate functional impairments even in individuals receiving suppressive therapy.

We observed that macrophages infected through direct contact with HIV-infected T cells displayed reduced viability even before osteoclast differentiation began. This increase in cell death was linked to inflammasome activation, as seen by higher levels of IL-1β production and partial recovery from caspase-1 inhibition. While pyroptosis is well-known in HIV-infected CD4^+^ T cells (27, 28), our findings extend this idea to macrophages. This reveals a new way that cell-to-cell HIV transmission triggers early inflammatory cell death in osteoclast precursors.

We further characterized these heterotypic interactions by noting increased physical contact between HIV-infected Jurkat cells and macrophages, accompanied by upregulated expression of adhesion molecules such as ICAM-1 and LFA-1, and tetraspanins including CD9, CD63, and CD81. These molecules are linked to forming virological synapses and viral egress (29–32) and in promoting HIV replication in macrophages through CD81-mediated, proteasome-dependent degradation of SAMHD1 (33). The observation that TAK-779-mediated CCR5 blockade reduced cell-to-cell contact supports the role of gp120–CCR5 signaling in stabilizing these interactions; however, the fact that infection efficacy was not affected suggests that additional mechanisms contribute to the infection process.

HIV-infected T cells showed a pro-inflammatory cytokine profile with higher levels of TNF-α and IFN-γ. These cytokines were released in larger amounts and concentrated where cells directly contacted each other. This process promoted M1-like polarization in macrophages. Such inflammatory changes can interrupt osteoclast differentiation by affecting RANKL signaling and reducing osteoclast-related genes (21, 22).

Another relevant finding is the disruption of redox balance. Although mitochondrial ROS (mROS) production is usually necessary for osteoclast differentiation, we noticed excessive ROS buildup in macrophages exposed to HIV-infected T cells. Pre-treating with NAC lowered both ROS levels and cell-to-cell contact. This suggests that HIV-related oxidative stress is not just a side effect of infection but also an active factor in intercellular interaction and resulting dysfunction. High ROS levels can hinder osteoclastogenesis by damaging key regulatory proteins and signaling pathways (34, 35), and may worsen this blockage under normoxic culture conditions, which are more oxidative than the natural bone marrow environment (36, 37).

Importantly, the negative effect of HIV on osteoclast differentiation depended on viral replication and the level of contact with infected T cells. Treatment with nevirapine helped restore osteoclast formation, and titration experiments indicated that even low levels of infected T cells could hinder osteoclastogenesis. These results match previous reports indicating that antiretroviral treatment, particularly drugs that target reverse transcription, can prevent HIV-related problems in bone precursor populations (8, 14).

In addition to the decrease in osteoclast numbers, we also noticed signs of function loss. This included issues with actin ring formation (38), lower integrin expression, reduced matrix adhesion, and a significantly lowered ability for bone resorption. Together, these data emphasize the complex nature of HIV-related osteoclast dysfunction, which involves viral replication, inflammation, oxidative stress, and blocked cell differentiation.

In summary, our findings support a model in which heterotypic HIV cell-to-cell transmission, particularly through T-cell contact, drives macrophage M1 skewing, mitochondrial reactive oxygen species (mROS) accumulation, and consequent impairment in osteoclast differentiation. Intracellular viral replication in macrophages is necessary for this dysfunction, as indicated by nevirapine reversing the defect, but alone may be insufficient unless infection kinetics and magnitude mimic the cell-to-cell transmission scenario. This mechanism, involving inflammatory and redox signaling, may contribute to increased bone loss and fragility in people living with HIV. Future research should explore the roles of specific viral proteins (e.g., Nef, Tat) and assess whether targeting inflammasome activation, oxidative imbalance, or intercellular adhesion pathways can preserve bone integrity in HIV infection.

While our study has strengths, it has limitations that need addressing. First, the model relies on *in vitro* co-cultures involving Jurkat cells and monocyte-derived macrophages. These may not fully represent the complex environments of lymphoid tissue or bone marrow, where tissue-resident macrophages interact with various immune and stromal cells. Second, Jurkat cells, while commonly used, differ from primary CD4^+^ T cells in activation status and cytokine production. Further validation using primary CD4+ T cells is deserved and planned. Third, our osteoclastogenesis tests were done under normoxic conditions, which can enhance ROS effects and do not reflect the physiological low oxygen levels of the bone marrow niche. Finally, while we show that nevirapine reduces HIV-related osteoclast dysfunction, more studies are needed to clarify the roles of specific viral proteins and host signaling pathways involved. Addressing these limitations, especially through ex vivo or *in vivo* models, will be key to validating and applying our findings in clinical practice.

## Data Availability

The raw data supporting the conclusions of this article will be made available by the authors, without undue reservation.
