# Supplementary material for: Inflammatory and redox reprogramming of macrophages by HIV cell-to-cell transmission inhibits bone resorption capacity
Source: Front Immunol. 2025 Nov 14;16:1694065. doi: 10.3389/fimmu.2025.1694065 (PMC12660246; doi:10.3389/fimmu.2025.1694065)
Supplement: Supplementary file 1 [file Table1.docx]

Supplementary Material

# Supplementary Figures and Tables

## Supplementary Figure 1


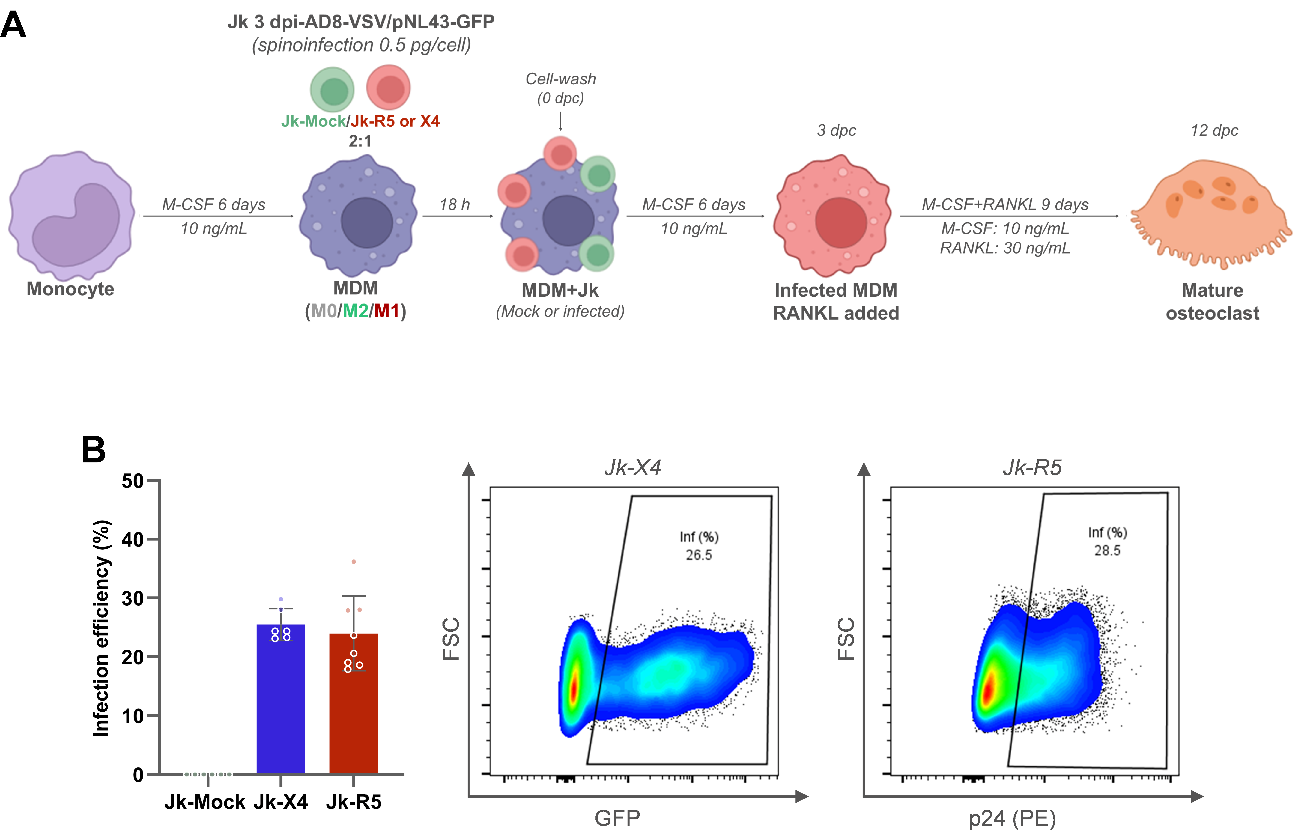


**Supplementary Figure 1.** **Experimental timeline and infection efficiency of Jurkat cells used for coculture. (A)** Schematic representation of the experimental design. Monocyte-derived macrophages (MDMs) were differentiated for 6 days with M-CSF (10 ng/mL), then co-cultured for 18 h with Jurkat cells previously infected for 3 days with R5- or X4-tropic HIV (via spinoculation at 0.5 pg p24/cell). After coculture, non-adherent cells were removed, and MDMs were further cultured for 3 additional days with M-CSF before being differentiated into osteoclasts by RANKL addition (30 ng/mL) for 9 days. **(B)** Infection efficiency (% GFP⁺ for X4 or % p24⁺ for R5) of Jurkat cells used for coculture, assessed at 3 dpi by flow cytometry. Representative dot plots for each HIV strain are shown. Each dot represents a biological replicate.

**
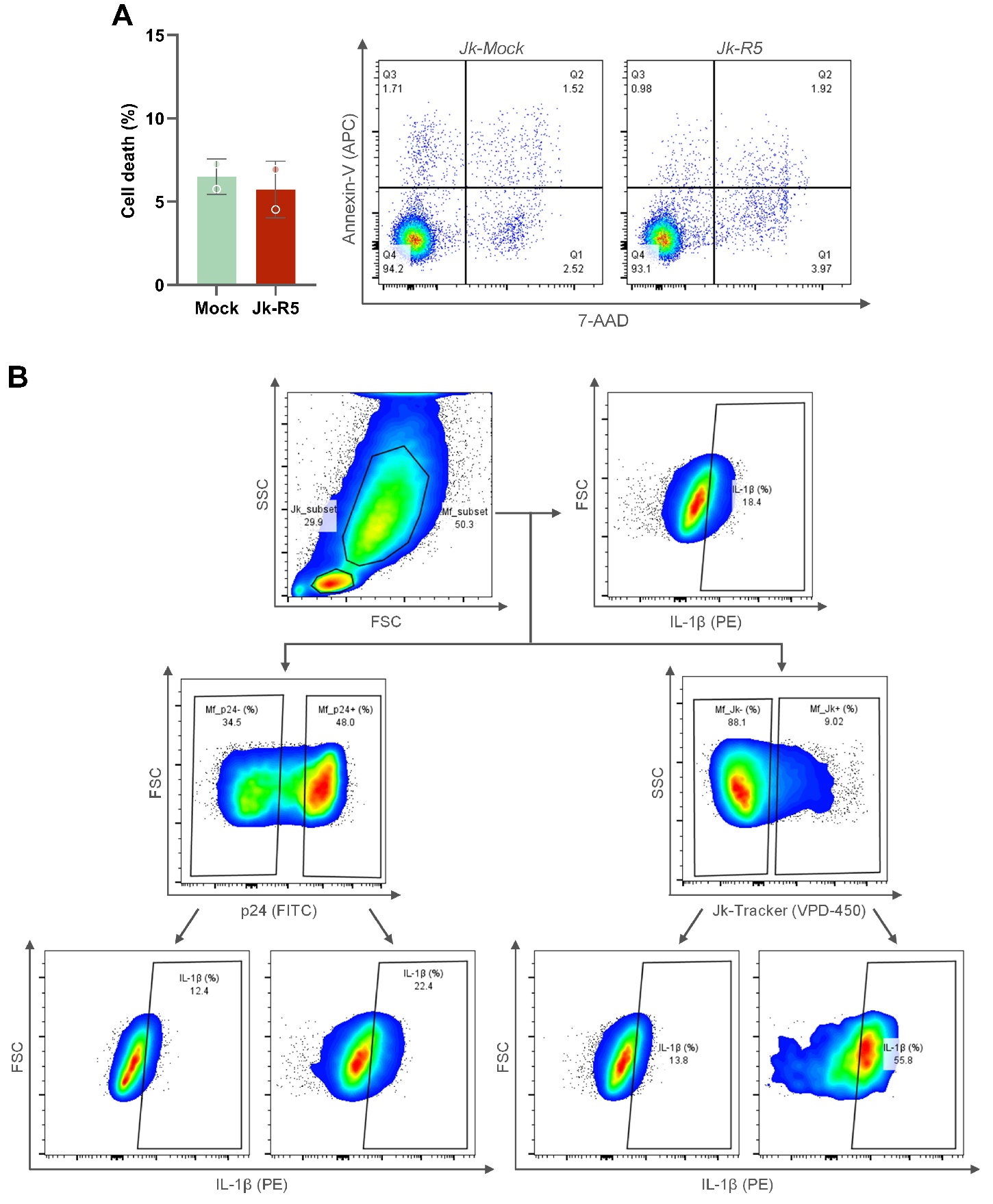
1.2 Supplementary Figure 2.**

**Supplementary Figure 2. Effects of HIV infection on Jurkat cell viability and gating strategy for IL-1β assessment. (A)** Cell death levels in Jurkat cells infected with HIV (Jk-R5) or mock-infected controls were evaluated at 3 dpi by flow cytometry using Annexin V/7-AAD staining. Bar graphs (top) and representative dot plots (bottom) are shown. Each dot represents a biological replicate. **(B)** Gating strategy used to assess IL-1β expression in macrophages after coculture. Jurkat and macrophage subsets were first identified by size. Within the macrophage gate, cells were further discriminated by p24 expression and Jurkat contact (positive for VPD-Jurkat tracker). IL-1β production was quantified in each subset using PE-conjugated anti-IL-1β staining.

**
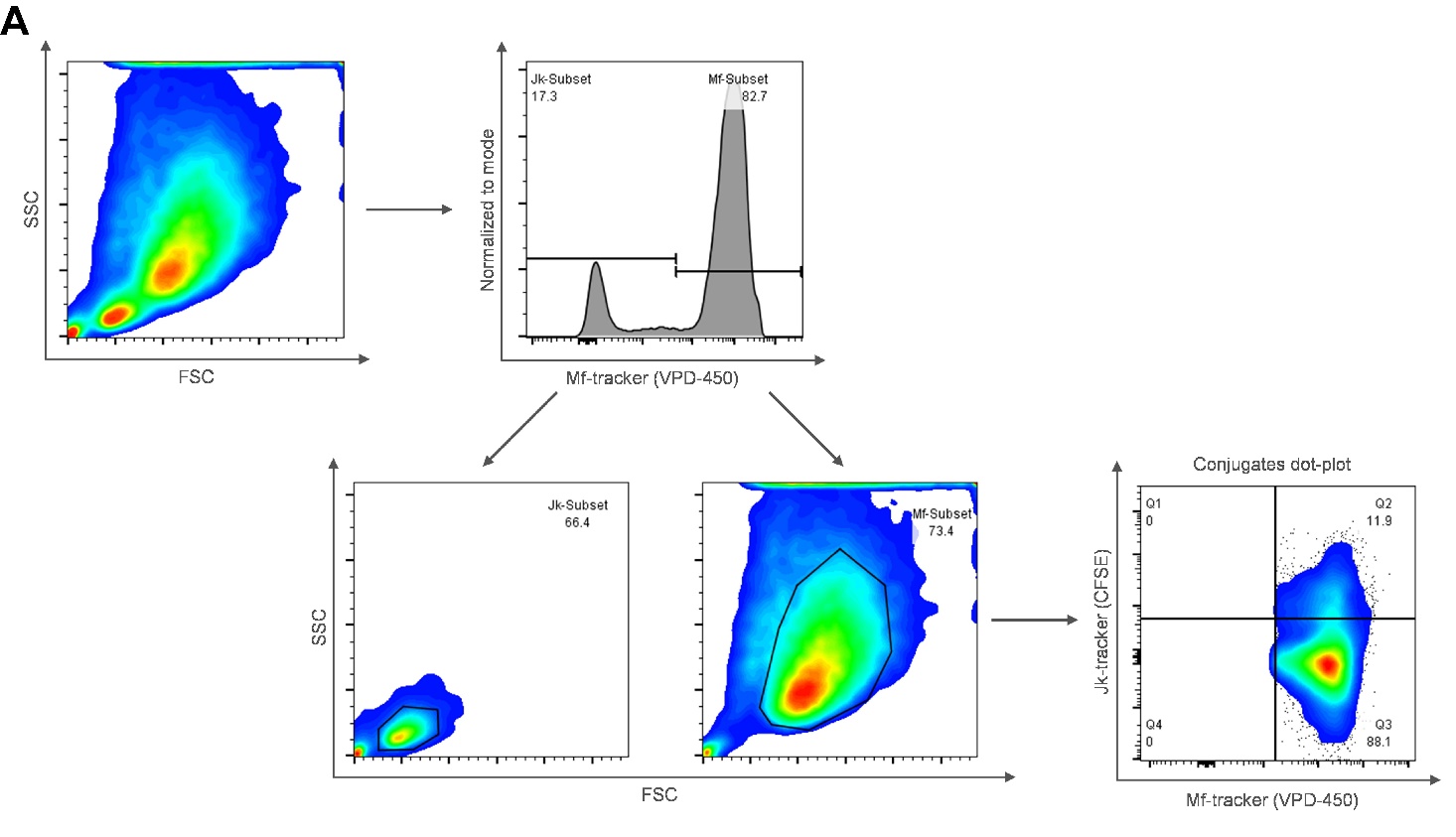
Supplementary Figure 3**

**Figure S3: Gating strategy for identification of macrophage–Jurkat conjugates by flow cytometry. (A)** Jurkat cells and macrophages were differentially labeled with CFSE and VPD450, before coculture. Total events were first separated into Jurkat and macrophage subsets based on VPD450 intensity. Each subset was then gated by FSC/SSC to refine the population. Double-positive events (CFSE⁺VPD450⁺) were identified as macrophage–Jurkat conjugates and quantified as a percentage of total macrophages. A representative gating strategy is shown.


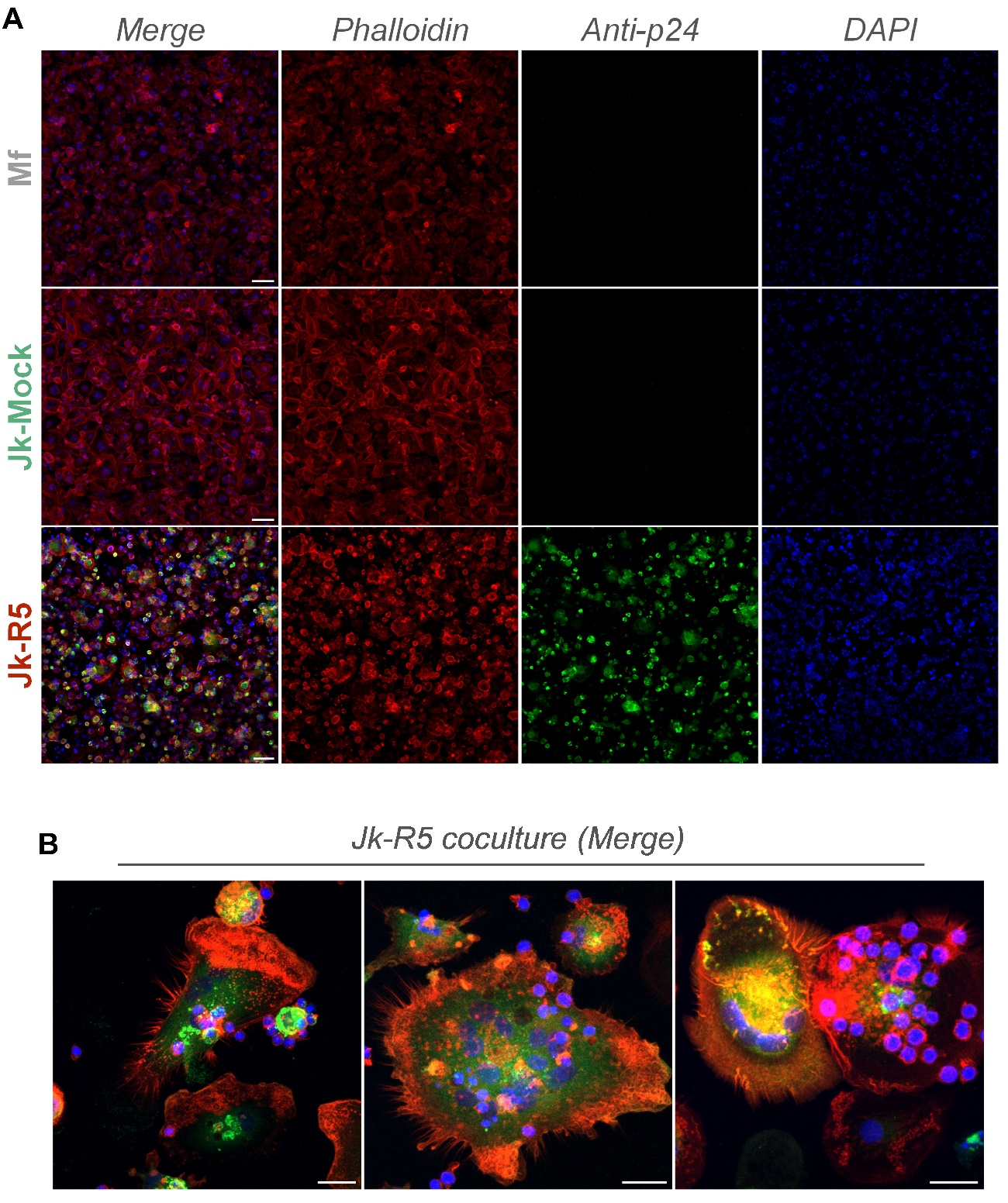
**Supplementary figure 4**

**Supplementary Figure 4. Additional confocal images of macrophage–Jurkat cocultures showing cytoskeletal changes upon HIV exposure. (A)** Representative immunofluorescence images of macrophage-only cultures (Mf), or cocultured with mock-infected (Jk-Mock) or HIV-infected Jurkat cells (Jk-R5), stained for F-actin (phalloidin, red), p24 antigen (green), and nuclei (DAPI, blue). Images were taken at low magnification to compare global cytoskeletal architecture and infection patterns across conditions. **(B)** Additional merged confocal images (z-projected) of selected HIV-exposed macrophages at higher magnification, highlighting disrupted actin ring formation and intracellular p24 accumulation. Scale bars = 100 μm (A), 25 μm (B).

**Supplementary Table 1. Primer Sequences**

| **Gene** | **Sequence (5’ to 3’)** |
| --- | --- |
| **IFN-γ**  Forward  Reverse | GAAACGAGATGACTTCGAAAAAGCTGA  CTGCTGGCGACAGTTCAGCCAT |
| **TNF-α**  Forward  Reverse | GTTCGAGAAGATGATCTGACTGCC  AGGCGGTGCTTGTTCCTCA |
| **IL-13**  Forward  Reverse | TGAGGAGCTGGTCAACATCA  CAGGTTGATGCTCCATACCAT |
| **TGF-β**  Forward  Reverse | GGACACCAACTATTGCTTCAG  TCCAGGCTCCAAATGTAGG |
| **GAPDH**  Forward  Reverse | CTCTGACTTCAACAGCGACAC  AGCCAAATTCGTTGTCATAC |
